# Gut–Brain Axis as a Pathological and Therapeutic Target for Neurodegenerative Disorders

**DOI:** 10.3390/ijms23031184

**Published:** 2022-01-21

**Authors:** Alma Rosa Lezama Toledo, Germán Rivera Monroy, Felipe Esparza Salazar, Jea-Young Lee, Shalini Jain, Hariom Yadav, Cesario Venturina Borlongan

**Affiliations:** 1 Center of Excellence for Aging and Brain Repair, Department of Neurosurgery and Brain Repair, Morsani College of Medicine, University of South Florida, 12901 Bruce B Downs Blvd, Tampa, FL 33612, USA; alma.lezamato@anahuac.mx (A.R.L.T.); german.riveramo@anahuac.mx (G.R.M.); felipe.esparzas69@anahuac.mx (F.E.S.); jeayoung@usf.edu (J.-Y.L.); 2 Center for Microbiome Research, Department of Neurosurgery and Brain Repair, Morsani College of Medicine, University of South Florida, 12901 Bruce B Downs Blvd, Tampa, FL 33612, USA; jains10@usf.edu (S.J.); hyadav@usf.edu (H.Y.)

**Keywords:** neurodegeneration, microbiome, stem cells, epigenetics, neurological disorders

## Abstract

Human lifestyle and dietary behaviors contribute to disease onset and progression. Neurodegenerative diseases (NDDs), considered multifactorial disorders, have been associated with changes in the gut microbiome. NDDs display pathologies that alter brain functions with a tendency to worsen over time. NDDs are a worldwide health problem; in the US alone, 12 million Americans will suffer from NDDs by 2030. While etiology may vary, the gut microbiome serves as a key element underlying NDD development and prognosis. In particular, an inflammation-associated microbiome plagues NDDs. Conversely, sequestration of this inflammatory microbiome by a correction in the dysbiotic state of the gut may render therapeutic effects on NDDs. To this end, treatment with short-chain fatty acid-producing bacteria, the main metabolites responsible for maintaining gut homeostasis, ameliorates the inflammatory microbiome. This intimate pathological link between the gut and NDDs suggests that the gut-brain axis (GBA) acts as an underexplored area for developing therapies for NDDs. Traditionally, the classification of NDDs depends on their clinical presentation, mostly manifesting as extrapyramidal and pyramidal movement disorders, with neuropathological evaluation at autopsy as the gold standard for diagnosis. In this review, we highlight the evolving notion that GBA stands as an equally sensitive pathological marker of NDDs, particularly in Alzheimer’s disease, Parkinson’s disease, amyotrophic lateral sclerosis and chronic stroke. Additionally, GBA represents a potent therapeutic target for treating NDDs.

## 1. Introduction

Neurodegenerative diseases (NDDs) are commonly defined as pathologies that lower normal brain function, usually accompanied by brain tissue atrophy and lower cognition capacity with a tendency to worsen with chronicity [1,2] Although NDDs manifest as chronic and aging brain pathologies, the exact timing for a brain pathology to turn into a neurodegenerative aberration remains not well understood. Acute brain insults with chronic pathological manifestations, such as stroke, present symptoms of neurodegenerative disease [3]. NDDs stand as a major health problem that affect millions of people worldwide, including those suffering from Alzheimer’s disease (AD), Parkinson’s disease (PD) and Amyotrophic Lateral Sclerosis (ALS), the most common pathologies around the world [4,5]. Most NDDs tend to be associated with aged people’s brain pathologies, and with the constant increase in life expectancy, the incidence of these diseases is expected to increase as well [1,4].

Over the past decade, the accumulating evidence has implicated a pathological link between brain and gut microbiota, which may also represent a novel therapeutic target for treating NDDs [6,7]. The gut-brain axis (GBA) is the term used to describe the interaction between brain and gut microbiota, highlighting the complex interaction of brain development, aging, and functioning [7,8]. Direct anatomical and physiological connections of the GBA may entail the vagus nerve, hormone signaling, metabolism of specific molecules such as tryptophan and even the immune system, altogether corresponding to key pathways for interrogating the GBA microbiome [6,9]. GBA may play an important role in brain functions, such as cognition learning and memory, suggesting that targeting the patients’ specific gut microbiota may alleviate neurological symptoms in NDDs, such as AD and PD [8,10]. Indeed, dampening the pro-inflammatory immune response product of pathogenic bacteria secretions and natural immune response in the gut affords therapeutic effects in NDDs [11].

Current medical treatment for NDDs is primarily symptomatic. Specific drugs that target the brain under a regimen of polypharmaceutical therapies can retard the progression of NDDs, but most of these pharmacotherapeutics are palliative and do not directly alter the disease pathology [6,12]. Novel therapies designed to promote disease-modifying outcomes, such as stem cell therapy, generate promising preclinical and clinical outcomes in NDDs [13,14]. Of note, several in vitro and preclinical models of NDDs reveal the mechanistic action of this stem cell therapy involves downregulation of the deleterious pro-inflammatory response closely associated with NDDs [14,15]. Recognizing the close interaction of inflammation in GBA and NDDs, in this article, we discussed the pathological hallmarks of NDDs (AD, PD, ALS, and chronic stroke), emphasizing the role of the gut microbiome in the disease progression as well as in developing innovative treatment via anti-inflammatory strategies, such as stem cell therapy. Many paradigms implicate the association of neurodegenerative diseases with infectious diseases and the putative biological function of some of the primary proteins implicated in these disorders. The aberrant accumulation of specific proteins, such as TDP-43 [16] and SOD1 [17] in ALS, amyloid β [18] and Tau [19] in AD, and α-synuclein [20] in PD, with similar proteinopathies seen in the chronic stage of ischemic stroke [21] can initiate a cascade of deleterious innate immunity processes that may contribute to the dysbiotic and dysfunctional GBA pathophysiological manifestations of NDDs. Recent review papers highlight the interaction between GBA and NDDs, with emphasis on diet, exercise, prebiotics and probiotics as treatments towards maintaining healthy microbiota in GBA [22,23,24,25,26,27]. Here, we chose to probe the underexplored use of stem cells for improving the microbiota in NDDs. While ethical and technical caveats abound, such as stem cell source, cell purity and amplification, and potential tumorigenic risks, hinder stem cell therapy, the optimization of the safety and efficacy of this approach may open new avenues of research and clinical application of cell-based therapeutics for treating NDDs.

## 2. Epigenetics and Neurodegeneration

Early human developmental stages represent a key period that may affect health in adulthood [28,29,30]. Indeed, a differentiation process takes place during the first 1000 days of life that leads to specialized cells of the pluripotent mediated by an epigenetic remodeling that is responsible for deactivating unnecessary genes for a particular tissue while helping to express those that are essential for the same [28]. To differentiate cells, DNA methylation processes dependent on DNA methyltransferases (DNMT) are necessary, which catalyze the methylation of CpG islands in the gene promoter. When the presence of methyl groups obstructs the interaction between the transcription factors and the promoter region, the binding with RNA polymerases that initiates early gene expression is suppressed [28,29,30]. An additional gene expression regulation through a complex process entails methylation of regulatory regions as well as histone methylation. The availability of methyl group donors determines the methylation process during pregnancy and throughout life via the folate metabolism pathway. A diet rich in folates, with folic acid supplementation during pregnancy, and the availability of vitamins B6 and B12 help to promote a good availability of methyl groups. In addition to DNA methylation, post-translational modifications and histone modification also stand as highly relevant epigenetic mechanisms associated with regulating a healthy and an unhealthy phenotype [28,29,30,31].

Chromatin remodeling is associated with the activation or inhibition of gene expression through processes such as histone methylation, acetylation, phosphorylation, ubiquitination, sumoylation, and glycation, altogether representing the first step for gene expression [28]. For the modulation of these processes, specific precursors are required for surveillance of adequate quantity and quality of nutrient intake to maintain a balanced ratio of FAD/FADH2. A reduced intake of folates during early life has been linked to incorrect DNA methylation with long-term effects, such as a decrease in the insulin growth factor 2 (IGF2) promoter methylation of the maternal allele transmission to offspring, overweight in men at age 20 and glucose intolerance at age 50 [28]. Additionally, low birth weight, obesity with coronary heart disease, and deterioration in neurocognitive development in adult life, with maternal smoking exacerbating this phenotype [28,29,30,31].

Neurological disorders, especially those presenting with neurodegeneration, have been associated with harmful environmental factors identified in childhood; in particular, an unbalanced diet that alters early gene expression leads to epigenetic changes that manifest in adulthood [28]. Early neurobehavioral deficits accompany remodeling of the epigenome by environmental factors such as smoking, alcohol, stress, and exposure to pesticides [32]. An iron deficit in early life is related to permanent deficits in recognition memory and, later in life, in procedural memory [33]. On the contrary, an excess of maternal iron or during adulthood can have deficiencies in development due to epigenetic and neuroinflammatory processes [34]. Studies in animal models showed that an iron deficiency in the neonatal age coincides with a neurodevelopmental dysfunction consequent with altered hippocampal DNA methylation and deficient expression of genes involved in the regulation of permeability, hypoxia, and angiogenesis [35]. A decrease in fetal neurogenesis may manifest as deficiencies in metals such as copper and zinc due to an impaired DNA methylation process, which during adulthood may contribute to the production of the β-amyloid peptide present in plaques of AD patients [28,29,30,31]. Similarly, dysfunctional fetal neurogenesis of immature dopaminergic neurons has been implicated in PD [20], whereas in ALS cellular modeling, induction, but not inhibition, of inflammation in fetal brain-derived human neural stem cells enhances their proliferation and differentiation into oligodendrocytes [36]. Interestingly in stroke, lactation protects the maternal brain against ischemic insult partly through angiogenic and neurogenic remodeling processes [37]. Altogether, these findings suggest that epigenetics in early life, or when recapitulated during pregnancy, may play a significant role in adult health, specifically regulating the brain capacity to undergo repair or neuroregeneration.

### 2.1. Amyotrophic Lateral Sclerosis

ALS, also known as motor neuron disease, manifests as a multifactorial neurodegenerative disease characterized by progressive degeneration and death of motor neurons in the brain and spinal cord, which leads to both motor and extra-motor symptoms. [38,39]. ALS diagnosis often occurs during the third and fourth decade of life and can be classified as sporadic, accounting for 90% of the cases, or hereditary, accounting for 10% of the cases [40]. Clinical features typically include muscle weakness, dysarthria, dysphagia, and, in more advanced stages, respiratory problems due to diaphragm paralysis [39,41].

The clinical neurodegeneration observed in ALS consists of decreasing ability to control and activate skeletal or smooth muscles, eventually manifesting as muscle weakness and wasting [42]. This muscular dysfunction is due to a loss of neuromuscular connection as well as axonal retraction, which leads to cell death of both upper and lower motor neurons [42,43]. Clinical symptoms can start with bulbar symptoms, such as dysarthria and dysphagia or can manifest in muscles of the extremities. Independent of mechanistic triggers of symptom initiation, neurodegeneration proceeds, and motor neuron death progresses to a point where patients are not self-sufficient anymore and need external help for basic life chores such as moving and eating [44]. Unfortunately, most of the patients die due to breathing or eating inability produced by near-complete incapacitation of breathing or swallowing muscles [43,44]. Despite the severity of motor neuron death, patients do not display cognitive or mental dysfunctions, thereby relegating ALS as a purely motor neurodegenerative disease.

**Figure 1:**
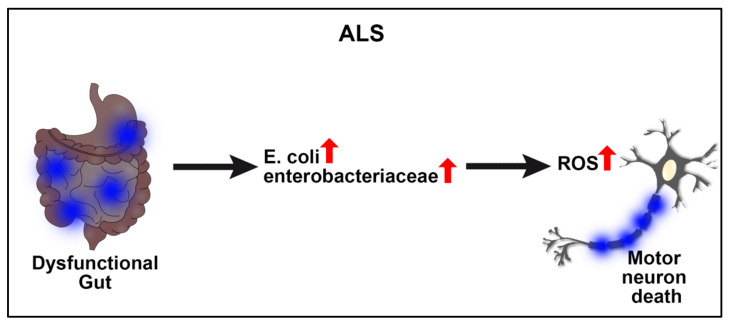
ALS and GBA. A dysfunctional gut accompanies the progression of ALS, with increased bacteria, including *E. coli* and enterobacteriaceae, leading to upregulation of damaging reactive oxygen species (ROS) and eventually contributing to motor neuron death, which is a hallmark pathological manifestation of the disease.

Although ALS etiology remains not well established, the gut microbiota may mediate the disease pathology, mainly due to pro-inflammatory gut microbiomes [45,46] (Figure 1). Of note, the gut pro-inflammatory state leads to neural disturbance, and with time, to neurodegeneration [46]. Pro-inflammatory cytokine expression contributes to the progressive damage of the central nervous system (CNS) and inhibits self-repairing processes [47,48]. Likewise, a deleterious feedback loop ensues with the initial inflammatory insult, subsequently triggering pro-inflammatory immune components such as microglia, macrophages, neutrophils, and natural killers to encroach cerebral tissue and create neurological dysfunctions [49,50].

Microglial activation acts as a major element of chronic neurodegeneration [51,52]. Patients suffering from ALS have higher levels of pro-inflammatory cytokines and biomarkers in cerebrospinal fluid and the spinal cord, such as IL-8, IL-6, MCP-1, and the expression of CD1, CD40, among others [53,54]. Pathologic bacteria expression of LPS and inflammatory cytokines, which are commonly associated with dysbiotic gut microbiomes, exacerbates chronic microglial activation [55,56]. Accordingly, an unhealthy gut state can lead to several neurologic disbalances, such as neurovascular unit (NVU) disturbance, blood–brain barrier (BBB) leakage, neurotoxic environment, etc., which together increase the risk to develop NNDs, such as ALS [46,53]. Upregulation of specific pathologic bacterias, such as E. coli and other enterobacteriaceae, accompanies ALS clinical manifestations and a poorer prognosis for long-term survival [53,57]. That the GBA may be the source of ALS pathophysiology is recognized from patients, as well as animal models, in their inability to eliminate reactive oxygen species (ROS) and other neurotoxic agents, which consequently increases motor neuron death [58,59]. This specific pathologic characteristic of ALS, in addition to the pro-inflammatory state produced by gut dysbiosis, creates a health scenario where it is crucial to attend to both the clinical neurological manifestations as well as the imbalance that exists at the gut level.

### 2.2. Alzheimer’s Disease

Advanced age is the main risk factor for AD. The composition of the intestinal microbiota changes as we age, and certain protective bacteria, such as Bacteroidetes, Bifidobac, and Lactobacillus, decrease [9,60,61]. The intestinal microbiota contains large amounts of bacterial amyloid, and the most studied is Escherichia Coli. The production of amyloid proteins prompts bacterial cells to form biofilms that confer resistance against the destruction of immune factors [62,63,64,65]. Exposure to bacterial amyloid proteins in the gut enhances the immune response to endogenous neuronal amyloid accumulation in the brain [11,66]

AD entails a complex neurodegenerative process that involves the aberrant formation of amyloid-β (Aβ) plaques as well as hyperphosphorylated Tau neurofibrillary tangles, thereafter producing neurotoxicity and neuroinflammation and resulting in cell death and lowering normal brain functions [67,68]. AD patients suffer from neuronal loss, specifically from the middle and lower temporal lobes, as revealed by imaging studies such as CT scans and MRIs [68]. This cellular death coincides with clinical manifestations such as lower semantic and episodic memory, which with time can make a person not self-sufficient for living alone or socializing with people [68,69]. The clinical onset of AD neurodegeneration usually starts with patients having difficulty remembering places, words, or names that they used to know [70]. Likewise, they have problems with learning new things or concepts and maintaining focus on a specific chore [71]. As neurodegeneration progresses, the symptoms worsen, and patients may not recognize familiar faces, places, or objects, recent activities, or known concepts, leading to behavioral changes [72,73].

**Figure 2:**
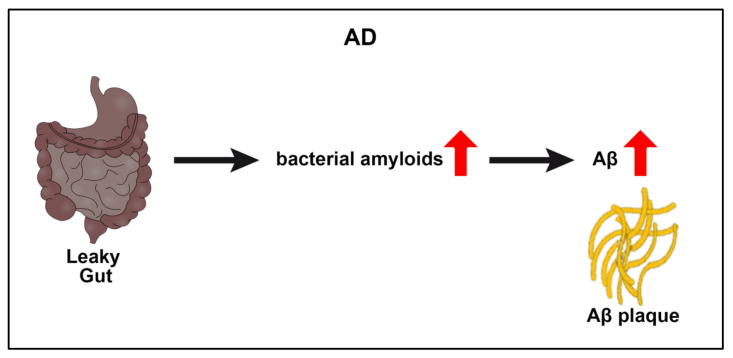
AD and GBA. A leaky gut may allow the transport of bacterial amyloids from the intestines to the brain, where aberrant aggregation of amyloid β (Aβ) occurs, forming Aβ plaques implicated in AD pathology and symptoms.

The pathogenesis of AD coincides with dysfunctional intestinal microbiota (Figure 2). Irritable bowel syndrome characterized by an alteration of the microbiota is one of the main pathophysiological factors of AD [74]. The bacteria that invade the intestinal microbiome have the ability to excrete huge amounts of amyloids and lipopolysaccharides, which can contribute to AD pathology [75]. Moreover, because AD is an age-related disorder, the BBB and the epithelium of the gastrointestinal tract become more permeable during aging, thereby allowing polysaccharides and amyloid to access the brain, easily causing inflammation [9,76]. Such age-induced compromise of the BBB and the gut suggests that the GBA may participate in the initial stages of AD-associated proteinopathy and inflammation.

### 2.3. Parkinson’s Disease

PD corresponds to the most common movement disorder affecting up to 1% of the population over 60 years of age. PD neurodegeneration etiology remains unclear, but neurotoxicity appears to arise from combined genetics and epigenetics alterations. The hallmark pathology of PD entails the depletion of dopaminergic cells located in the pars compacta of the midbrain [50,77]. Dopaminergic cell death leads to a dysfunction of dopaminergic pathways, mainly the nigrostriatal pathway, which is responsible for movement control [78]. Major clinical symptoms include resting tremor, bradykinesia and rigidity [79,80]. As neurodegeneration progresses, patients can experience mood and behavioral changes and limited facial movements and physical activity [81,82]. By the time clinical symptoms are clearly evident, it is estimated that around 80% of dopaminergic neurons have been lost [81].

**Figure 3:**
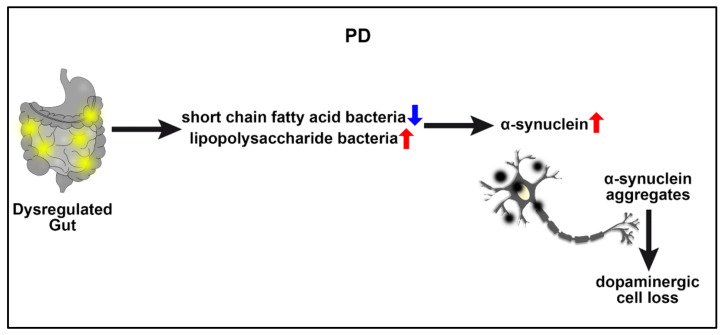
PD and GBA. Prior to dopaminergic depletion in the brain and even before the manifestation of PD symptoms, preclinical and clinical evidence indicates a dysregulated gut characterized by downregulated short-chain fatty acid bacteria but upregulated lipopolysaccharide bacteria, resulting in abnormal accumulation of α-synuclein in the gut that subsequently aggregates in the brain and causes dopaminergic degeneration, a PD pathological hallmark.

The association of the microbiome with PD is of particular interest since a healthy and dysbiotic microbiome can influence gut and brain homeostasis through complex two-way communication along the GBA [83,84] (Figure 3). The intestinal microbiome, largely affected by the diet, serves as a source of disease pathology but also represents a therapeutic target in preventing, modifying, or stopping PD [83]. A change in the gut composition of transgenic PD mice reveals GBA’s role in the pathogenesis of the disease since α-synuclein aggregates easily spread upward from the enteric nervous system to the brain [84]. Similarly, the components of the diet are closely related to the risk of suffering from PD since patients with this disease show a dysregulated intestinal microbiome (dysbiosis) characterized mainly by the loss of short-chain fatty acid bacteria and an increase in lipopolysaccharide bacteria [85,86]. Downstream neurodegeneration-inducing mechanisms of an altered microbiota in PD models include aberrant activation of the NLRP3 inflammasome, impaired insulin resistance, and dysfunctional mitochondrial [84,87,88].

The classic motor symptoms of PD reflect the death of dopamine-generating cells in the substantia nigra, but a wide spectrum of nonmotor clinical manifestations, among which there is a loss of smell, alterations in the gastrointestinal, cardiovascular and urogenital systems also accompany the disease [89]. Interestingly, gastrointestinal dysfunction is present in more than 80% of people with PD patients, suggesting that a deficient GBA contributes significantly to its pathogenesis [90]. As noted above, a bidirectional communication in GBA may preclude an initial excessive stimulation of the systemic innate immune system due to the dysregulation of the gastrointestinal system or bacterial overgrowth. Such dysfunctional GBA subsequently compromises the BBB permeability, resulting in a systemic-to-CNS influx of inflammatory microbiome, α-synuclein, altogether inducing deleterious immune and inflammation responses and ultimately dopaminergic neurodegeneration [84,90]. Another very important point to consider in this GBA-induced neurodegenerative pathology is that intestinal bacteria are capable of synthesizing various neurotransmitters and neuromodulators that allow intracellular communication [90].

Bacterial colonization is closely associated with postnatal development, including the maturation of the immune, endocrine and neural systems. These processes are highly relevant for efficient CNS signaling [91,92]. Indeed, a dysfunctional GBA accompanies disorders characterized by stress, depression, anxiety, irritable bowel syndrome, inflammatory bowel disease, and neurodevelopmental disorders, such as autism [90]. Of note, a significant percentage of PD patients present with symptoms, such as abnormal salivation, dysphagia, nausea, constipation, and impaired defecation, altogether corresponding to bodily functions associated with the gut [93,94]. Although a decrease in brain dopamine may mediate some gastrointestinal symptoms, peripheral organs (i.e., gut) are likely involved in the non-motor pathophysiology of PD [90]. The incorrect folding of α-synuclein is rampant in the intestinal microbiota of PD animals and accompanies the peripheral damage in dopaminergic neurons [95]. In parallel, the eradication of *H. pylori* in PD animals improved the absorption of the levodopa and reduced the motor symptoms [96,97]. In PD patients, worsening of motor severity proceeds with an infection of *H. pylori* [97]. Taken together, these findings suggest the close interaction of GBA in PD pathology and treatment.

### 2.4. Stroke

Stroke stands as the fifth cause of sudden death in the US. Common risk factors for stroke include arterial hypertension, smoking, age, and obesity [98,99]. This pathology involves thrombosis, embolism, or focal hypoperfusion that leads to cerebral blood flow interruption and consequent ischemia [100,101]. Lack of blood supply leads to cellular death, excitotoxicity, and an immediate pro-inflammatory response characterized by macrophages type 1 and T cells infiltration, the release of pro-inflammatory chemokines, oxidative stress, and reactive oxygen species production [102,103]. If blood reperfusion is not quickly restored or the pro-inflammatory environment is not sequestered, it can lead to severe complications such as BBB disruption and critical neuronal loss with considerable brain functional disability [104].

While traditionally considered an acute injury, stroke manifests with chronic neurodegeneration. A stroke consists of two key pathological events [105]. The first one involves the initial injury and death of neurons due to ischemia. Cell loss in the initial injury cannot be recovered, and, depending on the anatomical location of the ischemia, specific clinical symptoms ensue [15,105]; frontal lobe ischemia can lead to motor dysfunction, while temporal lobe ischemia may induce language and speech deficit as well as memory and cognitive impairment [106]. The second stroke event entails a neurodegenerative event likely mediated by microglial activation, BBB leakage, oxidative stress, chronic inflammation, among other cell death mechanisms [15,107]. Our long-standing interest in chronic neuroinflammation reveals that this cell death process can be present weeks or even months after the ischemic event and can enhance late neurodegeneration [15,107,108]. Similar to acute stroke symptoms, chronic stroke symptoms associated with neurodegeneration may vary depending on the anatomical region of the ischemia, but some of the most commonly reported manifestations include dizziness, amnesia, disorientation, and constant headache [108,109]. Treating acute stroke, as well as chronic stroke (albeit), neuroinflammation may need enhanced post-ischemic patient medical care.

**Figure 4:**
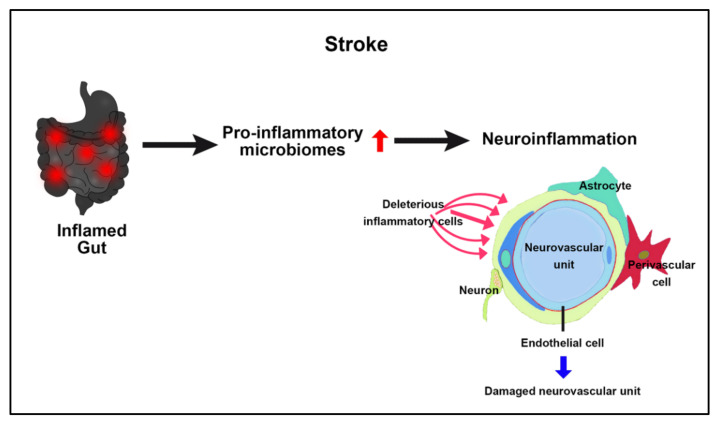
Stroke and GBA. Following the initial primary injury of ischemic injury (acute phase), the gut mounts an inflammatory response, resulting in the production of deleterious pro-inflammatory microbiomes, which, when uncontrolled over time (chronic phase), leads to detrimental inflammation that damages the neurovascular unit, thereby exacerbating stroke outcomes.

Probing the role of GBA bidirectional communication in stroke reveals cell death pathways [110] (Figure 4). After ischemia, damage-associated molecular patterns (DAMPs) not only trigger cerebral inflammation but also induce a gut inflammatory response [111]. Gut inflammation can lead to intestinal injury, increased gut permeability, and even sepsis [112]. Furthermore, gut inflammation confers systemic inflammation that contributes to brain inflammation [110,113]. Pro-inflammatory gut microbiomes accompany a worst stroke prognosis likely due to a heightened immune system that generates a detrimental pro-inflammatory response after cerebral ischemia [110,114]. Reminiscent of established neurodegenerative disorders, such as ALS, AD, and PD, as discussed above, the significant contribution of GBA to stroke secondary injury requires a closer examination of this cell death pathway in the stroke pathology and its treatment.

Current stroke treatments, such as tissue plasminogen activator (tPA) and mechanical thrombectomy, are highly timing-dependent after the stroke onset in order to be effective [115]. The therapeutic window of 4.5 h and 24 h for tPA and mechanical thrombectomy [116,117,118,119] are limited to treating the acute stroke injury, i.e., restoration of blood supply. However, cognizant of the chronic stroke complications, specifically the neurodegeneration, novel treatment strategies need to be developed to improve stroke clinical outcomes.

## 3. GBA-Based Stem Cell Therapy for NDDs

Treatment options for neurological diseases are very limited and mostly palliative instead of disease-modifying therapies. Stem cell therapy represents a breakthrough in abrogating the neurodegenerative disease process owing in large part to the regenerative features of the stem cells that recapitulate brain development [120]. Indeed, the application of stem cell therapy in neurological diseases has reached clinical trials based on solid safety and efficacy data over the last three decades [121]. Stem cells have unique properties, among which are their capacity for self-renewal, differentiation, and growth factor secretion, which by themselves can initiate the regenerative process or stimulate the host brain to foster brain repair [121].

There are several sources for stem cells, such as the fetus, embryo, and adult tissues such as bone marrow, adipose, placenta, and umbilical cord, among others, each with promising applications [122,123,124]. Pluripotent stem cells appear to confer multi-pronged regenerative processes, including neural differentiation and by-stander growth factor effects, which may afford disease-modifying outcomes, especially in neurodegenerative diseases and ischemic processes [15,124,125,126]. Moreover, the wide window (e.g., several days to weeks and even many months after disease diagnosis) in transplanting stem cells in animal models and patients with NDDs circumvents the narrow treatment intervention timing seen with tPA and mechanical thrombectomy in the case of stroke.

Animal models of NDDs have shown an improvement in neural function after the transplantation of different cells or their derivatives by replacing lost neural cells, releasing cytokines, modulating inflammation, and mediating remyelination, among other regenerative mechanisms [127,128,129,130]. Although preclinical and clinical studies show the safety of transplanting stem cells, whether directly into the brain or peripherally via intravenous or intra-arterial routes, the demonstration of efficacy remains elusive for a number of factors but primarily due to optimization of cell dose and timing.

Recognizing that the pathology of NDDs encompasses not just brain degeneration but early aberrant alterations in the gut point to a novel strategy for transplanting stem cells in these neurological disorders. In particular, targeting the stem cells to the gut rather than the brain may be more practical and effective in the view that gut dysbiosis precedes neurodegeneration. We, and others, have shown that many intravenously administered stem cells in PD animal models preferentially migrated into the gut than the brain [131,132]. Moreover, this preferential gut migration of the stem cells reduced inflammatory microbiota and dampened inflammation in both gut and brain [131,132]. Such GBA-targeting of stem cells has also been explored in ALS, in that reducing the microbial burden in mutant mice by transplanting gut microflora from a protective environment suppressed harmful systemic and neural inflammation produced by gut dysbiosis even at the ALS symptomatic period [133]. Our studies provide further evidence that the microbial composition of our gut has an important role in brain health and can interact in surprising ways with well-known genetic risk factors for disorders of the nervous system. In AD, while directly transplanting stem cells into the gut remains to be tested, a similar concept of treating gut dysbiosis with healthy microflora specifically with *B. bifidum* BGN4 and *B. longum* BORI effectively blocked amyloidosis and apoptotic processes, enhanced synaptic plasticity, and reduced cognitive and memory impairment in AD mice [134]. Finally, in strokes, we advance a similar GBA-focused stem cell therapy whereby we highlight that peripheral inflammatory responses accompany strokes, necessitating a paradigm shift from purely central towards incorporating peripheral sequestration of cell death pathways to improve stroke therapeutic outcomes [135,136,137,138]. The body of evidence from basic and clinical investigations of NDDs suggests that the underlying homeostatic and pathophysiological functions of GBA represents a novel approach in advancing our knowledge of the disease pathology and treatment, i.e., cell-based regenerative medicine needs to consider GBA-targeted treatments.

## 4. Conclusions

A better understanding of the GBA could provide novel perspectives of NDD pathophysiology and therapeutic approaches. Profiling of the microbiome signature of specific NDDs may reveal distinct microbiota associated with gut dysbiosis. In the same token, these microbiota may serve as therapeutic targets for treating NDDs. To this end, an inflammatory microbiome closely approximates NDD progression, and dampening this harmful microbiome retards neurodegeneration. In particular, transplanting stem cells into the gut of preclinical models of NDDs reduces the inflammatory microbiome not just in the gut but also in the brain accompanied by improvement in neurological functions. Whereas the present paper focuses on just four NDDs, other neurological disorders present with similar GBA alterations that accompany the disease progression, including Huntington’s disease [139,140,141] and multiple sclerosis [142,143,144]. Accordingly, disease-specific tailoring of stem cell transplantation targeting GBA may provide disease-modifying outcomes for these neurological disorders. The fact that the GBA plays a significant role in disease pathology advances the innovative concept of GBA-based therapeutics for NDDs.

## Data Availability

This manuscript is a review paper based on literature review, thus no primary data are available.
